# The efficiency paradox of discharge masking head loss in run-of-river hydropower generation

**DOI:** 10.1038/s41598-026-36906-3

**Published:** 2026-01-22

**Authors:** Mugaruka Josue Mugisho, Bayongwa Samuel Ahana, Vithundwa Richard Posite, Sophie Ngayirwa, Derrick Mirindi, Frederic Mirindi, Cherifa Abdelbaki, Navneet Kumar

**Affiliations:** 1 Centre des Recherches en Environnement et Géo-Ressources, Université Catholique de Bukavu, Bukavu, Democratic Republic of the Congo; 2 Institute of Water and Energy Sciences Including Climate Change , Pan African University University of Tlemcen , B.P. 119, Tlemcen, 13000 Algeria; 3 Department of Water and Forest , Official University of Semuliki Democratic Republic of the Congo , Beni city, Democratic Republic of the Congo; 4 Institut Supérieur Pédagogique de Bukavu (ISP Bukavu) Laboratoire de Physique Des Solides et Des Interfaces (LPSI), Bukavu, Democratic Republic of the Congo; 5 School of Architecture and Planning , Morgan State University , Baltimore, MD USA; 6 School of Engineering and Applied Sciences, Pennsylvania, USA; 7 Department of Economics , University of Manitoba , Manitoba , Canada; 8 EOLE laboratory , University of Tlemcen , Tlemcen, 13000 Algeria; 9 Division of Ecology and Natural Resources Management, Center for Development Research (ZEF), University of Bonn, Genscherallee 3, Bonn, 53113 Germany

**Keywords:** Hydropower efficiency, Operational resilience, Sedimentation, Head loss, Run-of-River, Machine learning, Sustainable energy management, Engineering, Environmental sciences, Hydrology

## Abstract

While climate impacts on hydropower output are well-documented, plant efficiency, the critical ratio of electrical energy generated to hydraulic energy input, remains an underexplored metric, particularly in data-limited regions. This study analyzes the efficiency dynamics of the Ruzizi I plant (29.8 MW) from 2000 to 2023 to unravel the interplay between hydrological drivers and operational constraints. Building on the established context of a hydraulic trade-off between water volume and head, we employed machine learning (Multiple Linear Regression, Random Forest, Gradient Boosting) and operational analysis to diagnose efficiency drivers. Results reveal that plant efficiency increased significantly (+ 3.6%-points/decade) and is overwhelmingly governed by discharge (*r* = 0.998), with machine learning models confirming the negligible role of head and seasonality. This indicates that efficiency gains are almost entirely flow-dependent, masking the potential negative impact of head loss. The system exhibits strong buffering from Lake Kivu, with efficiency remaining stable during drought but surging by 17–18% during wet years. Crucially, operational analysis identified an optimal load factor range (78–82%) that could improve efficiency by ~ 4% points compared to historical operation. However, a concurrent decline in available capacity factor (− 5.5%/decade) signals emerging non-hydrological constraints. These findings underscore that while water volume currently dominates efficiency gains, long-term sustainability requires managing sediment-induced head loss and optimizing operations within the identified optimal range to mitigate the underlying vulnerabilities in the energy conversion process.

## Introduction

Hydropower constitutes a foundational element of the global renewable energy portfolio, essential for climate change mitigation and sustainable development goals. In Africa, its role is even more critical, providing the bulk of electricity generation in many nations and forming the backbone of regional energy integration initiatives^1^. However, the continent’s hydropower infrastructure faces a dual challenge: increasing variability in climatic drivers and the pervasive aging of its physical assets. While a substantial body of research has effectively documented the sensitivity of hydropower output to climate variables such as precipitation and temperature^2,3^, a more fundamental metric, plant efficiency, has received considerably less attention. Efficiency, defined as the ratio of electrical energy generated to the hydraulic energy input, serves as the ultimate indicator of a plant’s conversion health, reflecting the interplay of turbine performance, head utilization, and operational management^4^. Consequently, while energy production measures the final product, efficiency unveils the underlying performance of the conversion process itself, offering a more nuanced lens through which to assess the long-term viability and resilience of hydropower assets.

Most climate–hydropower studies quantify impacts on energy production or capacity by linking generation to precipitation, temperature, and drought conditions^2,3^. However, plant production efficiency, the ratio of electrical output to hydraulic energy input, is less frequently examined despite its usefulness for diagnosing conversion performance and separating hydrological forcing from operational or mechanical limitations^4^. Accordingly, large-scale assessments and some energy–water modelling frameworks often represent efficiency as a constant parameter, potentially overlooking gradual changes driven by sediment-related head losses, equipment wear, and evolving operating regimes^5–7^. In parallel, the hydropower engineering literature has developed methods to infer or adjust unit efficiency characteristics from measured power–head–discharge data (e.g., Hidalgo et al., 2014), but these approaches are rarely connected to climate-impact analyses that typically emphasize output metrics over conversion dynamics.

For run-of-river schemes, the efficiency signal can be particularly difficult to interpret because gains in turbined discharge may coincide with declining gross (or net) head due to tailwater rise, sediment deposition, or downstream geomorphic adjustments^8^. Recent work also underscores that this is not a purely statistical issue but a conceptual one: Bragalli et al.^9^ argue that performance evaluation of existing run-of-river plants is comparatively less debated, and show that net head fluctuations induced by processed flow rate are frequently neglected even though downstream discharge conditions and residual energy can significantly affect net head. To capture the combined influence of head variability and electromechanical efficiency, they introduce the concept of a plant “Global Efficiency” that links overall plant performance to both generation-group efficiency and the flow-dependent net head term, and demonstrate that the best efficiency point at plant scale may differ markedly from what is suggested by the generation-group efficiency curve alone. This conceptual framework motivates the need for plant-level diagnostics that explicitly track how joint variations in discharge and head shape observed efficiency, especially in systems where long-term head loss may be masked by increasing discharge.

Empirical evidence that jointly quantifies these competing hydraulic trends and their net effect on long-term efficiency remains limited, particularly in data-limited African contexts, especially for systems buffered by large upstream lakes or reservoirs that dampen seasonal rainfall variability^10,11^. The Ruzizi River cascade, shared by Burundi, the Democratic Republic of Congo, and Rwanda, exemplifies these intertwined challenges, where transboundary water management intersects with aging hydropower infrastructure and land–water–energy trade-offs^12,13^. Ruzizi I, commissioned in 1958, has recently been shown to exhibit a notable hydraulic trade-off: increasing water availability has supported higher generation, while gross head has declined persistently over time. This combination raises an important performance question that cannot be resolved from production data alone: what is the net effect of increasing discharge conditions and declining head on the plant’s energy conversion efficiency? In run-of-river and sediment-influenced systems, reduced head and hydraulic losses can erode conversion performance even when flows are sufficient, and operators may partially compensate by shifting operating points or relying on higher turbined discharge^8^. Yet, efficiency is rarely evaluated explicitly in many hydropower assessments, which often prioritize output metrics over the conversion process that reflects turbine condition, abrasion, and operational optimization^4^. Without quantifying efficiency dynamics, it remains unclear whether recent gains in generation reflect genuinely improved performance or whether they partly mask underlying efficiency losses linked to head reduction and technical constraints.

To address these questions, this study conducts an efficiency-centric analysis of the Ruzizi I hydropower plant using monthly operational records from 2000 to 2023. Following the plant-level (global) efficiency concept^9^, our goal is to attribute observed efficiency variability to routinely measured hydraulic and operational controls. Specifically, we aim to (1) characterize long-term trends and seasonal variability in operating production efficiency; (2) quantify the relative contributions of turbined discharge, gross head, and capacity availability using complementary statistical and machine-learning models; (3) assess efficiency responses during droughts and wet years; and (4) identify operating ranges associated with higher efficiency to inform plant management. This framework provides an empirical basis for interpreting efficiency signals in aging, sediment-influenced run-of-river systems and for distinguishing whether higher generation reflects improved conversion performance or compensation for deteriorating head conditions.

## Methodology

### Study area

**Fig. 1:**
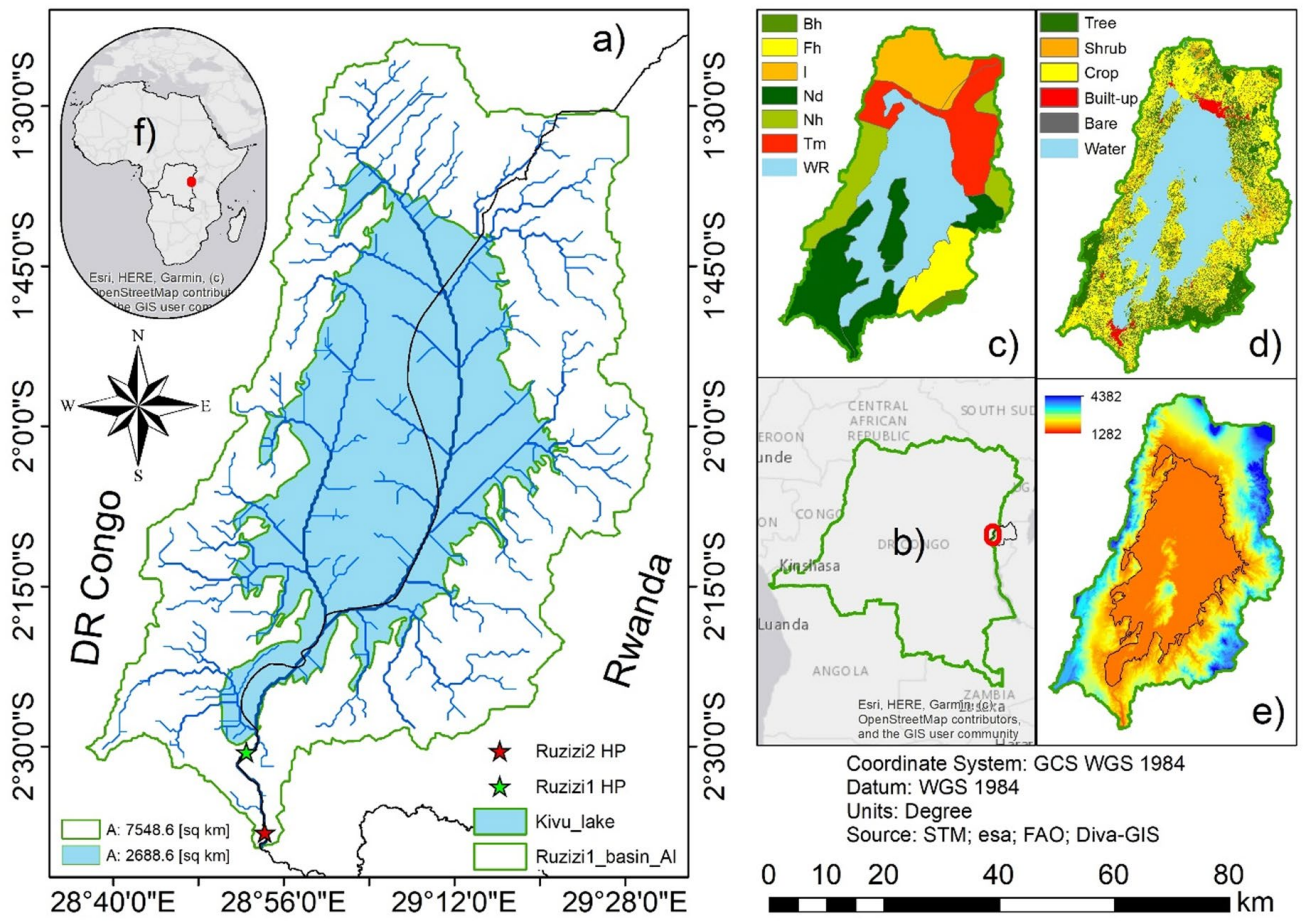
Study area of the Ruzizi I hydropower plant. (**a**) Area of interest (AOI), (**b**) location within the DRC and Rwanda, (**c**) soil map, (**d**) land use/land cover (LULC) map, (**e**) digital elevation model (DEM), and (**f**) regional location in Africa.

The Ruzizi River Basin (RRB) extends over 12,802 km² in the Great Lakes region of Central Africa, spanning the territories of Rwanda, Burundi, and the Democratic Republic of the Congo (DRC). Geographically, the basin lies between − 1.397283° and − 3.368295° latitude, and 28.623113° and 29.539972° longitude. Originating in Lake Kivu, the Ruzizi River flows southward into Lake Tanganyika, delineating parts of the international border between Rwanda and the DRC^14^. The present study focuses specifically on the controlled catchment of Ruzizi I, which regulates river flow along the Rwanda–DRC border (Fig. 1). This sub-basin is central to hydropower operations, providing a controlled water supply that underpins regional energy generation. The hydrological and geological setting is strongly influenced by the East African Rift System, characterized by Cenozoic volcanic rocks, Precambrian gneisses, and sedimentary formations, with active volcanism and recurrent seismic activity shaping the landscape and posing challenges for infrastructure development.

Hydropower development is the dominant human use of the Ruzizi River. Ruzizi I, commissioned in 1959^15^, has a catchment area of 32 km², a reservoir capacity of 1.46 million m³, an installed capacity of 29.8 MW, and uses Kaplan turbines, making it the longest-serving hydropower facility in the basin. Kaplan turbines are commonly used in low-head/run-of-river contexts because adjustable blades and guide vanes maintain relatively high efficiency across a range of flows^16^. Downstream, Ruzizi II, operational since 1989, draws from a 90 km² catchment with a reservoir volume of 1.75 million m³, producing approximately 43.8 MW of electricity^12,13,17^. Together, these plants form a transboundary energy lifeline for the region. Looking ahead, major expansions are planned. Planned future projects include Ruzizi III and Ruzizi IV, both of which remain under development. Ruzizi III is the most advanced, with design plans envisioning a 224 km² catchment area and a 1.9 million m³ reservoir, while Ruzizi IV is still at the early planning stage. Once realized, these facilities are expected to markedly expand the basin’s hydropower capacity, enhance flow regulation along the transboundary river, and reinforce energy security for Rwanda, Burundi, and the DRC^13^.

### Data and methods

**Fig. 2:**
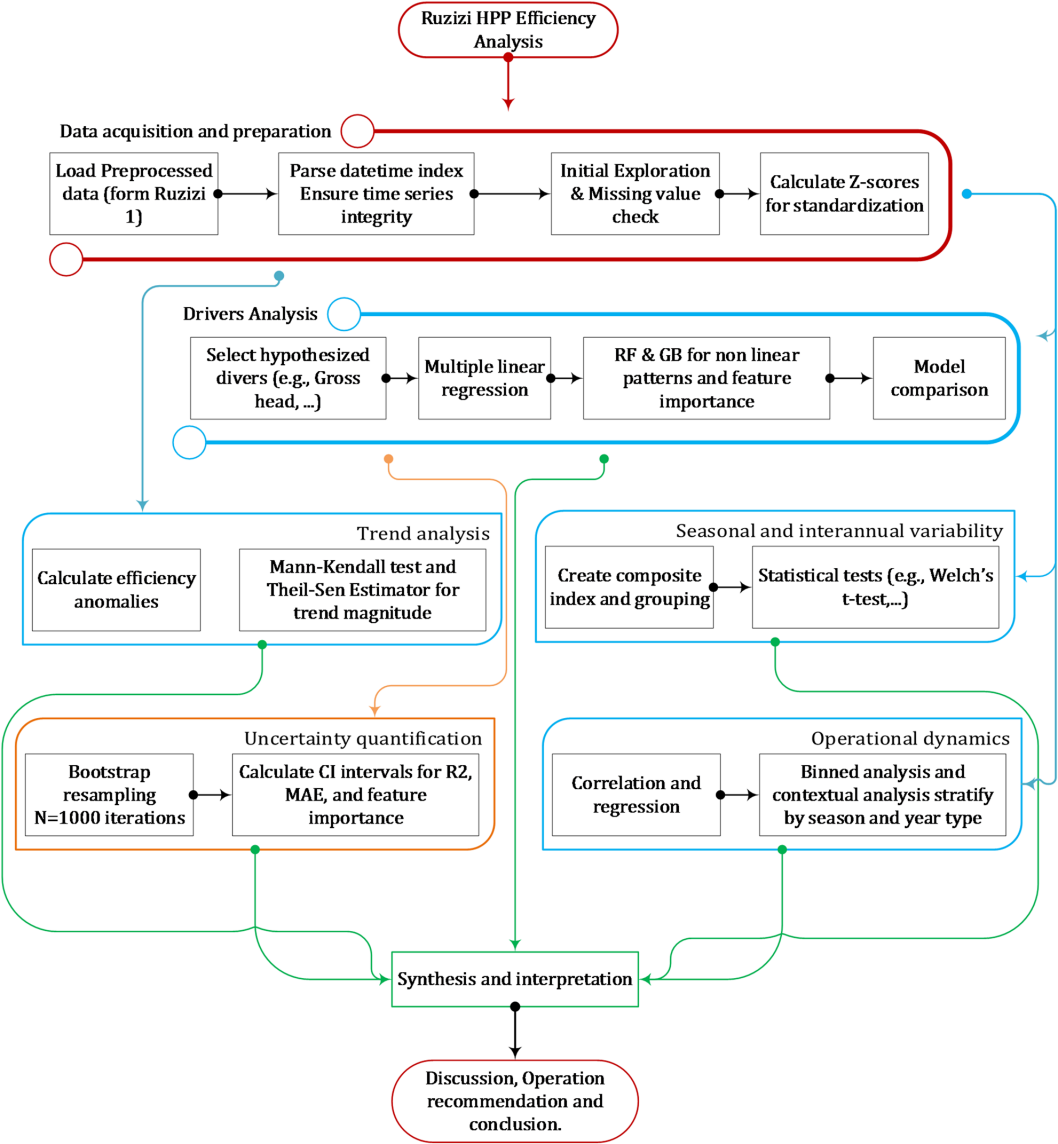
Flow chart of the methodology.

This study employed a structured framework to examine long-term efficiency dynamics at the Ruzizi I hydropower plant through sequential and interconnected analyses. The process began with data acquisition and exploratory analysis to establish dataset integrity and baseline characteristics. Next, trend analysis identified significant monotonic changes, followed by an assessment of seasonal and interannual variability using hydro-climatic classifications. A multi-model machine learning framework was then applied to identify key efficiency drivers, with bootstrap resampling used for uncertainty quantification. Finally, operational dynamics analysis evaluated relationships between efficiency and plant load/capacity utilization under varying hydro-climatic conditions. The overall workflow is summarized in a methodological flowchart (Fig. 2), highlighting how each phase contributes to diagnosing efficiency patterns and drivers at Ruzizi I.

#### Data acquisition and preliminary exploration

The analysis commenced with the acquisition of the pre-processed operational dataset for the Ruzizi I hydropower plant, spanning January 2000 to December 2023 at a monthly resolution. The dataset was sourced from the national electricity utility (SNEL) and included comprehensive performance metrics: gross and net energy production (MWh); average and peak power output (MW); turbined water volume (m³) and discharge (m³/s); reservoir, barrage, and tailwater levels (m.a.s.l.); gross hydraulic head (m); plant efficiency (%); operating hours; and available and installed capacity (MW). These operational records were complemented by climate data from the ERA5-Land reanalysis^18^, a gridded dataset selected for its physical coherence, high spatial (0.1° × 0.1°) and temporal resolution, and proven reliability in hydrological and energy applications across data-sparse regions, providing a robust, gap-free climate record essential for trend analysis^19^.

Missing data imputation followed a hierarchical approach designed to preserve seasonal patterns and physical constraints. Primary gaps were filled using harmonic regression with Fourier terms to capture annual and semi-annual cycles^20^. For variables with insufficient data for harmonic regression, monthly climatological means and linear interpolation were applied as fallbacks^21^. Physical bounds (e.g., efficiency between 0 and 100%, non-negative precipitation) were enforced. Across 13 variables, 337 missing values were imputed, with monthly correlation coefficients between observed and imputed data averaging 0.996 (ranging from 0.974 for tailwater level to 0.999 for dam level and operating hours), confirming strong preservation of seasonal signals.

**Fig. 3:**
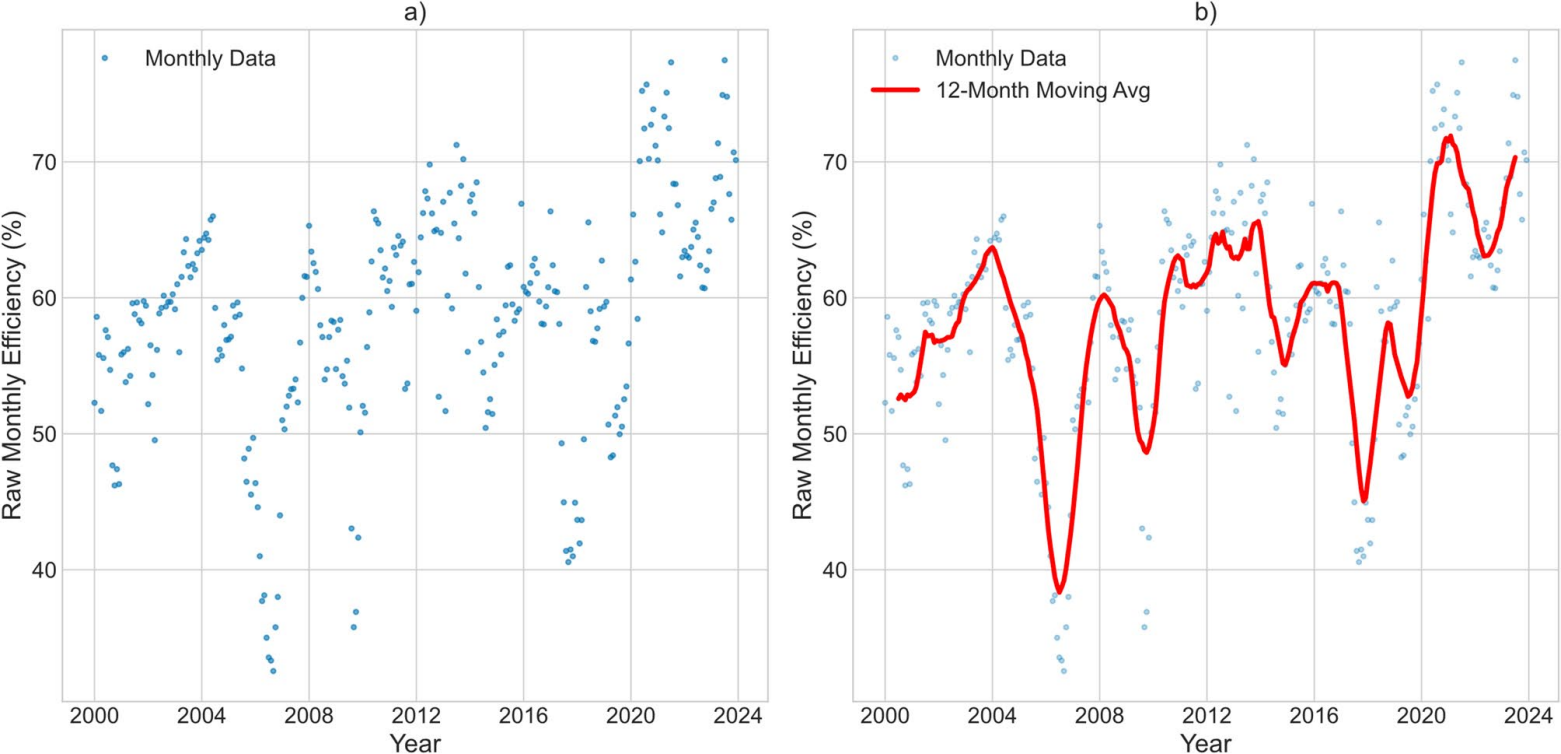
Long-term pattern of efficiency: **a**) Raw monthly efficiency; **b**) 12-month moving average.

The dataset was imported into a Python computational environment (v3.9+) utilizing the pandas library (v1.3.0) for data manipulation^22^. The Date field was parsed as a datetime object and set as the DataFrame index, a crucial step for ensuring the integrity of all subsequent time series analyses^23^. An initial exploratory data analysis (EDA) was conducted to assess data structure, completeness, and the distribution of the primary variable of interest: plant efficiency (Efficiency_pct). This involved inspecting dataset dimensions, data types, and generating summary statistics (mean, standard deviation, min, max, quartiles) to establish a baseline understanding of the data’s central tendency and dispersion^24^. To visualize the underlying long-term pattern in efficiency amidst monthly variability, a 12-month centered moving average was applied and plotted alongside the raw monthly data (Fig. 3). This smoothing technique is well-established for suppressing high-frequency noise and revealing lower-frequency trends and cycles in hydroclimatic time series^21,25^. The matplotlib (v3.4.0) and seaborn (v0.11.0) libraries were employed for visualization (Fig. 3), utilizing a seaborn-whitegrid style and a colorblind-friendly palette to enhance graphical clarity and accessibility.

In this study, we analysed the time-varying operating efficiency computed at each monthly timestep as

$$\:\eta\:\left(t\right)=P\left(t\right)/\rho\:g\hspace{0.17em}Q\left(t\right)\hspace{0.17em}H\left(t\right),$$.

where *P*(*t*) is measured power output, *Q*(*t*) is turbined discharge, *H*(*t*) is gross head, *ρ* is water density, and g*g* is gravitational acceleration^4,9^. This corresponds to the expected value of the monthly efficiency time series.

This differs from an aggregated global (or long-term average) efficiency, which would compute a single constant ratio of mean power to mean hydraulic energy input. The global formulation explicitly weights the covariance between *Q*(*t*) and *H*(*t*), whereas the time-varying definition allows us to diagnose month-to-month variability and long-term trends in the conversion process. Because our primary goal is to attribute drivers of monthly efficiency variability and to detect gradual changes in plant performance, we adopt the operating-efficiency definition. This choice clarifies that the “masking” of head loss by discharge variability discussed in our results is specific to the monthly operational signal, where head varies within a relatively narrow range and co-varies weakly with discharge.

#### Trend analysis methodology

To quantitatively assess long-term changes in plant efficiency, a trend analysis was conducted on monthly efficiency anomalies. Anomalies were calculated as the deviation of each monthly value from the long-term mean, effectively removing the mean state to focus on variability and change over time^21^. The efficiency anomaly $\:A\left(t\right)$ for each month $\:t$ was computed as:

$$\:A\left(t\right)=E\left(t\right)-\stackrel{-}{E}$$

Where; $\:E\left(t\right)$ is the efficiency percentage at time $\:t$, and $\:\stackrel{-}{E}$ is the mean efficiency over the entire study period.

The non-parametric Mann-Kendall (MK) test was employed to detect the presence of a monotonic trend in the anomaly time series^26,27^. This test is recommended for hydroclimatic data analysis due to its robustness against non-normal distributions and resilience to outliers, which are common in environmental datasets^28,29^. The test assesses whether there is a statistically significant monotonic upward or downward trend by calculating the Kendall’s tau (τ) statistic and its associated p-value. For the time series where the MK test indicated a significant trend (*p* < 0.05), the magnitude of the trend was quantified using the Theil-Sen estimator^30–33^. This robust non-parametric method calculates the median slope of all possible lines through pairs of points, making it resistant to up to 29% outliers in the data, a valuable property for long-term environmental monitoring^21^. The resulting slope was expressed both per month and per decade to enhance the interpretability of long-term changes^34,35^. The analysis was visualized by plotting the monthly anomalies, a 12-month moving average to elucidate low-frequency variability, and the Theil-Sen trend line. A histogram of the anomalies was also examined to assess their distribution around zero.

#### Analysis of seasonal and interannual variability

To assess variability across different timescales, the efficiency anomalies were analyzed for seasonal patterns and interannual variability, particularly during extreme hydro-climatic events.

- Classification of extreme hydro-climatic years.

A composite index approach was employed to objectively classify each year of the study period into ‘Drought’, ‘Normal’, or ‘Wet’ categories. This methodology follows established practices in hydroclimatology for identifying coherent drought and pluvial events that consider multiple hydrological variables^36,37^. The composite index $\:C{I}_{year}$​ for each year was constructed using two key indicators:

Where; $\:{Z}_{P,\:year}$ is the z-score of the annual cumulative precipitation total for a given year, and $\:{Z}_{L,\:year}$​ is the z-score of the annual mean Lake Kivu level for that same year. This equally weighted combination creates a holistic hydro-climatic index. Years were then classified based on established percentile thresholds:

- Drought years: $\:C{I}_{year}\le\:-1$ (approximately ≤ 10th percentile).
- Normal years: $$\:-1<C{I}_{year}<1$$
- Wet years: $\:C{I}_{year}\ge\:1$ (approximately ≥ 90th percentile).

b. Analysis of variability.

The seasonal cycle was analyzed by grouping efficiency anomalies by the predefined ‘Dry’ and ‘Wet’ seasons and calculating summary statistics (mean, standard deviation) for each. Interannual variability was assessed by computing the mean annual efficiency anomaly for each calendar year. To quantify the statistical significance of observed differences, a Welch’s t-test was used to compare efficiency anomalies between dry and wet seasons^38^. This test is appropriate for comparing two independent groups with potentially unequal variances, a common scenario in environmental data^39^. For the comparison across the three hydro-climatic year types (Drought, Normal, Wet), a one-way Analysis of Variance (ANOVA) was first conducted. Where the ANOVA indicated significant differences (*p* < 0.05), Tukey’s Honestly Significant Difference (HSD) post-hoc test was applied to identify which specific group pairs differed significantly^40^. This multi-step approach controls the family-wise error rate when making multiple comparisons^41^. The results were visualized using a combination of bar plots (for seasonal and annual means with error bars), box plots (to show distributions and outliers within seasons and extreme year categories), and statistical annotations.

#### Analysis of efficiency drivers

To identify and quantify the primary drivers of efficiency variations at Ruzizi I, a multi-model machine learning framework was employed. This approach combines interpretable linear models with powerful non-linear algorithms to provide a comprehensive understanding of the underlying relationships.

- Data preprocessing and feature selection.

All variables were standardized by converting them to z-scores to ensure comparability of effect sizes across different units and scales. The z-score for each variable $\:X$ was calculated as:

Where; $\:{\mu\:}_{x}$​ is the mean and $\:{\sigma\:}_{x}$​ is the standard deviation of variable $\:X$^42^. This transformation facilitates the interpretation of regression coefficients as the change in the outcome variable (in standard deviations) associated with a one standard deviation change in the predictor variable. The predictor variables were selected based on hydrological principles and plant operational characteristics: Gross_Head_m_z (hydraulic driving force), Discharge_m3s_z (water throughput), and Available_Capacity_MW_z (technical availability). The categorical variable Season was one-hot encoded for inclusion in the models^42^.

b. Modeling framework.

Three distinct modeling approaches were implemented to assess driver importance from complementary perspectives:

1. Multiple Linear Regression (MLR): Served as a baseline interpretable model. The standardized coefficients from MLR provide direct estimates of the unique contribution of each driver while controlling for others, representing the linear relationship between predictors and efficiency^43^.
2. Random Forest Regressor (RF): A powerful ensemble method that captures non-linear relationships and complex interactions between variables without requiring prior specification of these relationships^44^. Hyperparameter tuning was performed using grid search with 5-fold cross-validation to optimize model performance and prevent overfitting.
3. Gradient Boosting Regressor (GBR): An advanced boosting algorithm that sequentially builds an ensemble of weak predictors, often providing state-of-the-art predictive performance for tabular data^45^. Similar hyperparameter tuning was applied.

The dataset was split into training (80%) and testing (20%) sets to evaluate model performance on unseen data. Model performance was assessed using the coefficient of determination (R²), mean absolute error (MAE), and root mean squared error (RMSE). Feature importance was extracted from the tree-based models to quantify the relative contribution of each driver to the predictive accuracy.

c. Uncertainty quantification.

To assess the robustness and reliability of the model performance metrics and feature importance estimates, a comprehensive bootstrap uncertainty analysis was conducted. Bootstrapping is a powerful non-parametric resampling technique that allows for the estimation of sampling distributions and confidence intervals for virtually any statistic, making minimal assumptions about the underlying data distribution^46^. The bootstrap procedure involved generating B = 1000 pseudo-datasets (i.e., 1000 independent resampling iterations) by randomly sampling the original training data with replacement, each time fitting the three candidate models (Multiple Linear Regression, Random Forest, Gradient Boosting) and evaluating their performance on the unchanged test set. Because the dataset is at a monthly time scale, we note that a naive i.i.d. bootstrap could be inappropriate if there were substantial serial dependence. However, exploratory checks indicated no material serial autocorrelation in the monthly series. In addition, as a robustness check, we repeated the uncertainty analysis using a moving-block bootstrap with 12-month blocks (one annual cycle), and the resulting performance uncertainty estimates were essentially unchanged. This process creates an empirical distribution for each performance metric (R², MAE), from which 95% confidence intervals were calculated using the percentile method (2.5th and 97.5th percentiles). This approach provides a robust estimate of the uncertainty associated with each model’s performance due to sampling variability^47^. For the Random Forest model, the bootstrap procedure was extended to quantify the uncertainty in feature importance estimates. The variability in importance scores across bootstrap samples provides crucial information about the stability of the identified drivers; features with narrow confidence intervals around high importance values can be considered robust determinants of efficiency, whereas those with wide intervals indicate less stable relationships^48^. The statistical significance of performance differences between models was assessed by examining the distribution of pairwise differences in R² values across all bootstrap samples. The probability that one model genuinely outperforms another was calculated as the proportion of bootstrap iterations where this difference was positive, providing a Bayesian-like interpretation of model comparison^49^.

#### Analysis of operational dynamics

The final analytical phase examined the relationships between efficiency and key plant operational indicators to identify potential optimization opportunities. This investigation focused on two critical operational metrics: Load Factor (the ratio of average power to peak power, indicating how consistently the plant operates near its maximum capacity) and Available Capacity Factor (the ratio of average power to available capacity, reflecting how fully the available generating capacity is utilized). Pearson correlation coefficients were calculated to quantify the linear relationships between efficiency and both operational metrics. Simple linear regression was then employed to model these relationships, following the form:

$$\:Efficiency=\beta\:0+\beta\:1\times\:Operational\:Metric+\epsilon$$

Where; $\:\beta\:0$​ is the intercept, $\:\beta\:1$​ is the slope coefficient, and ϵ*ϵ* is the error term^43^. These analyses tested the hypothesis that operational strategies aimed at maximizing output (high load factor) might come at the expense of conversion efficiency.

- Binned analysis for optimal operating point identification.

To move beyond linear assumptions and identify potential non-linear relationships or optimal operating ranges, both load factor and available capacity factor were divided into 10 equal-frequency bins using the *pd.cut()* function. For each bin, the mean efficiency was calculated, allowing for the identification of operational ranges that maximize efficiency rather than simply maximizing output^42^. This approach is particularly valuable for identifying potential trade-offs between different performance objectives in complex engineering systems.

b. Contextual analysis by season and hydro-climatic condition.

Recognizing that optimal operations may vary under different conditions, operational metrics and efficiency were compared across seasons (Dry vs. Wet) and previously defined hydro-climatic year types (Drought, Normal, Wet). This stratification allowed for the examination of whether operational strategies should be adapted based on environmental conditions^3^.

The relationships were visualized through scatter plots with regression lines, bar charts showing efficiency by operational bins, and comparative bar charts across seasons and year types. These visualizations facilitated the interpretation of complex multivariate relationships and the communication of practical operational insights.

## Results

### Trend in anomalies

**Fig. 4:**
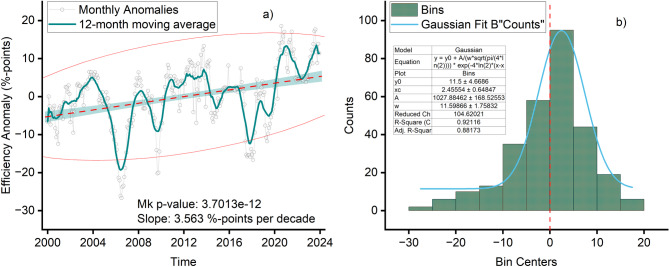
Trend in the data: (**a**) displays the long-term trend of efficiency anomalies together with the Theil–Sen slope and the 12-month moving average; (**b**) shows the distribution of efficiency anomalies.

The Mann–Kendall test revealed a statistically significant positive trend in efficiency anomalies over the study period (τ = 0.275, *p* < 0.001). This confirms that the improvements observed are not attributable to random variability but reflect a persistent directional shift in system performance. The Theil–Sen estimator further quantified the magnitude of this trend (Fig. 4a), yielding an average increase of 0.030%-points per month, equivalent to + 3.56%-points per decade. This represents a substantial rate of improvement, consistent with progressive long-term operational gains at the Ruzizi I hydropower station. In addition to the upward trajectory, the monthly distribution of efficiency anomalies was right-skewed, indicating that while negative deviations occurred, positive anomalies were both more frequent and more extreme (Fig. 4b. This suggests that efficiency improvements were not only gradual but also characterized by episodic jumps above the baseline, likely reflecting short-term operational adjustments or favorable hydro-climatic episodes superimposed on the long-term trend. The combination of significance testing and slope estimation establishes clear evidence of progressive efficiency gains across the 2000–2023 period. These gains form the foundation for subsequent analyses on the drivers of variability and the system’s responses under different hydro-climatic conditions.

### Seasonal & interannual variability

**Fig. 5:**
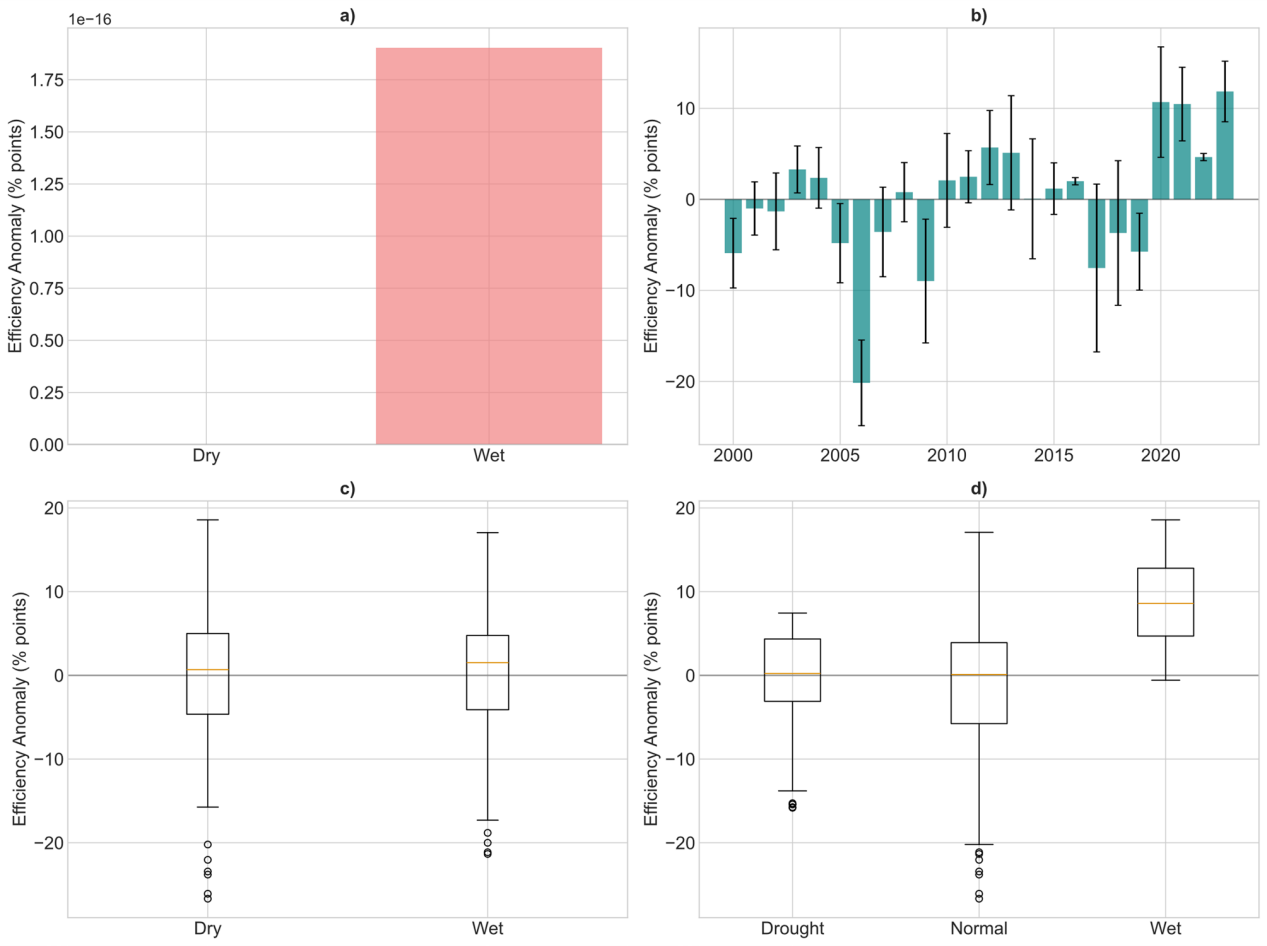
Seasonal and interannual variability of efficiency anomalies: (**a**) mean anomalies by season, (**b**) yearly means with standard deviations, (**c**) seasonal distributions, and (**d**) anomalies during extreme years.

The classification of hydro-climatic conditions using the composite index (precipitation + lake level) produced 216 normal months, 36 drought months, and 36 wet months over the 2000–2023 period, providing a balanced framework for comparing efficiency across contrasting hydrological regimes. On a seasonal scale, efficiency anomalies averaged 0.0%-points in both the Dry and Wet seasons, with comparable variability (Dry: σ = 9.13, Wet: σ = 7.68; Fig. 5a). A two-sample t-test confirmed that there was no significant difference in efficiency between Dry and Wet seasons (t ≈ 0.000, *p* = 1.000). This indicates that seasonal efficiency fluctuations are not strongly determined by intra-annual rainfall distribution, but are instead smoothed out by plant operations and the run-of-river system’s regulation capacity. At the interannual scale, efficiency anomalies displayed marked fluctuations across years (Fig. 5b). The largest negative deviations occurred in 2006 (−20.2%-points) and 2009 (−9.0%-points), both associated with dry or operationally constrained conditions. Conversely, the years 2020 (+ 10.7%-points), 2021 (+ 10.5%-points), and 2023 (+ 11.8%-points) recorded the strongest positive anomalies, coinciding with hydrologically favorable conditions and higher reservoir levels. These results highlight the sensitivity of plant efficiency to year-to-year variations in water availability and plant operation.

When analyzed by extreme hydro-climatic categories, drought and normal years showed similar efficiency levels (means of −1.46 and − 1.27%-points, respectively), whereas wet years were characterized by substantially higher efficiency anomalies (+ 9.06%-points; Fig. 5d). This pattern was strongly supported by statistical tests. An ANOVA revealed significant differences among classes (F = 29.36, *p* < 0.001), and post-hoc Tukey tests indicated that wet years differ significantly from both drought and normal years (*p* < 0.001), while drought and normal years remain statistically indistinguishable (*p* ≈ 0.99). Finally, the overall variability of efficiency anomalies was moderate, with a standard deviation of 8.3%-points and a full range spanning from − 26.7 to + 18.6%-points (Fig. 5c). This distribution confirms that while efficiency generally remains stable, hydrologically favorable years provide a distinct performance boost that is not mirrored by equivalent losses during drought years, suggesting an asymmetric system response.

### Drivers of efficiency

#### Deterministic drivers and model results

**Fig. 6:**
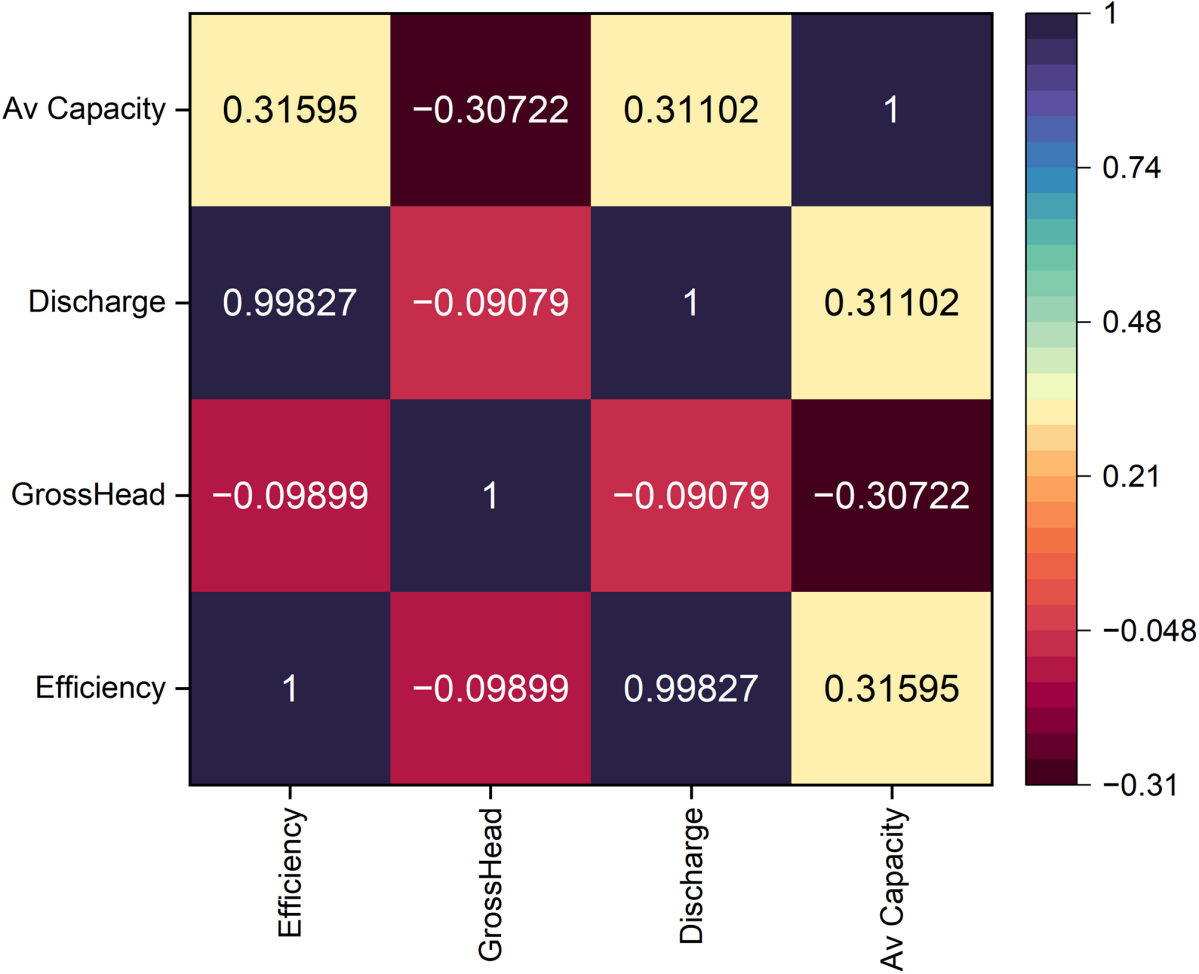
The correlation coefficients among variables, with color intensity and size indicating the strength and direction of the correlations.

Across the full monthly dataset (*n* = 288), the z-score correlation matrix (Fig. 6) indicates that efficiency (Efficiency_pct_z) is almost perfectly aligned with discharge (Discharge_m3s_z) (*r* = 0.998), while its association with gross head (Gross_Head_m_z) is weak and slightly negative (*r* = − 0.099). This near-perfect correlation between discharge and efficiency ($\:r=0.998$) was rechecked using de-trended anomalies, yielding $\:r\approx\:0.97$, confirming that the relationship is physical and not an artifact of mathematical coupling in the efficiency formula. The correlation with available capacity (Available_Capacity_MW_z) is modest and positive (*r* = 0.316). Among the predictors, gross head is weakly and inversely related to available capacity (*r* = − 0.307) and to discharge (*r* = − 0.091), whereas discharge and available capacity are modestly positively related (*r* = 0.311). These patterns point to a hierarchy in simple linear associations in which discharge dominates variance shared with efficiency, followed by a smaller contribution from available capacity and minimal co-variation with head.

**Fig. 7:**
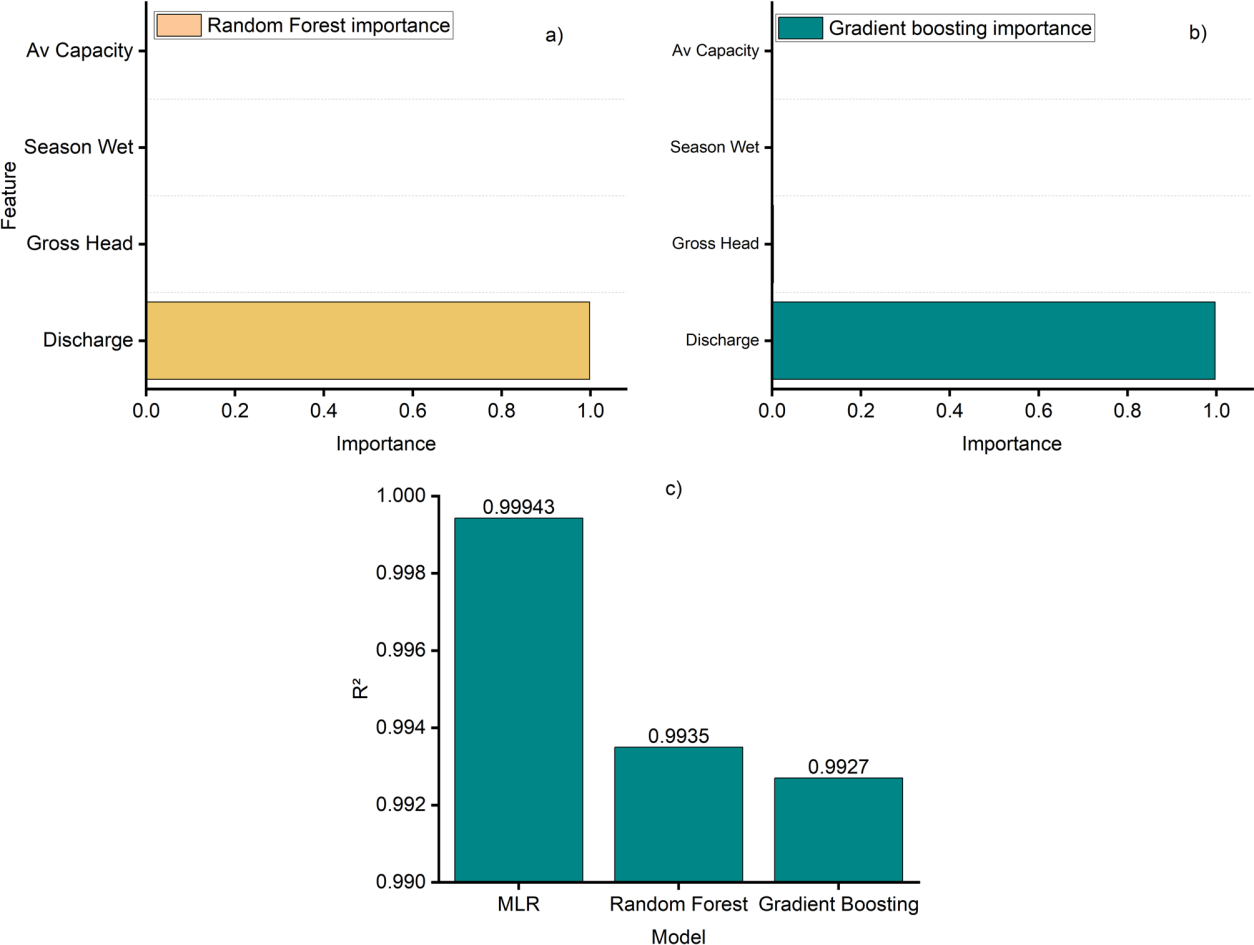
Model performance and feature importance. (**a**) Relative importance of predictor variables in the Random Forest (RF) model, (**b**) relative importance of predictor variables in the Gradient Boosting model, and (**c**) comparison of model performance metrics across machine learning approaches.

The multiple linear regression using standardized predictors (features: Gross_Head_m_z, Discharge_m3s_z, Available_Capacity_MW_z, and a seasonal dummy for Wet) reproduces this ranking in the partial effects (Table 1; Fig. 7c). The standardized coefficient on discharge is 0.950, far larger than those on gross head (− 0.010), available capacity (0.004), and the Wet-season indicator (− 0.001). On the held-out test set, the model achieves R² = 0.999 with very small errors (MAE = 0.015 z-units; RMSE = 0.025 z-units), indicating that, in this linear specification, nearly all of the explainable variance in efficiency z-scores is accounted for by the included predictors, with discharge providing the overwhelming contribution. Tree-based models corroborate this structure. The tuned Random Forest yields R² = 0.993 (MAE = 0.038; RMSE = 0.086) and assigns 99.8% of total importance to discharge, with only trace importance to gross head (~ 0.1%) and effectively none to the season indicator and available capacity. Gradient Boosting returns nearly identical performance (R² = 0.993; MAE = 0.040; RMSE = 0.091) and a nearly identical importance profile (discharge ~ 99.7%, gross head ~ 0.3%, season and available capacity ~ 0%).

**Table 1:** Performance metrics of the tested models, including MLR, RF, and GB. Metrics include mean R² and mean MAE across bootstrap samples, standard deviations, and 95% confidence intervals, providing a comprehensive quantification of model uncertainty.

Model	*R*²_mean	*R*²_std	*R*²_CI_lower	*R*²_CI_upper	MAE_mean	MAE_std	MAE_CI_lower	MAE_CI_upper
MLR	0.9993	0.0004	0.9984	0.9996	0.0160	0.0064	0.0101	0.0312
RF	0.9921	0.0039	0.9836	0.9975	0.0436	0.0077	0.0297	0.0576
GB	0.9922	0.0047	0.9814	0.9986	0.0432	0.0106	0.0260	0.0599

Thus, across linear and non-linear learners, the ranking of predictors is consistent: discharge is the primary driver of monthly efficiency variability in standardized units, followed by negligible incremental contributions from gross head, available capacity, and season (Fig. 7a, b). Taken together, the correlation structure, standardized regression coefficients, and model-based importance scores all converge on the same quantitative ordering. Discharge explains almost all of the observed month-to-month variance in efficiency z-scores in this dataset, while head, available capacity, and seasonal regime contribute comparatively little within the tested specifications and feature set. Residual errors are uniformly small across models, and the driver ranking is stable to the change in modeling framework.

#### Model comparison under uncertainty

**Fig. 8:**
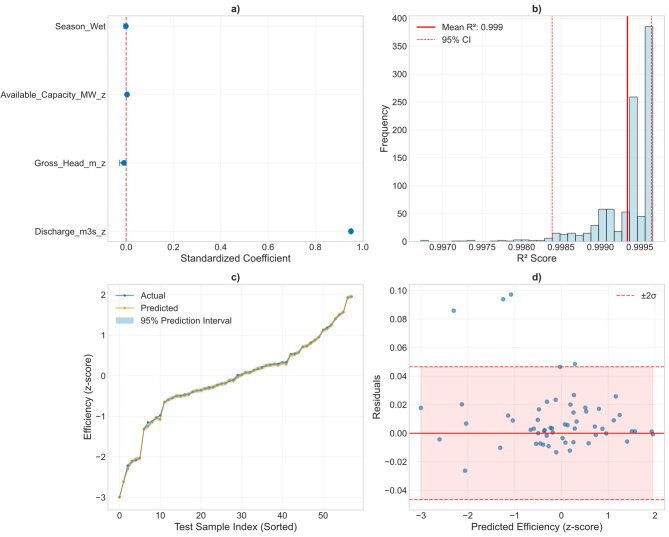
**a**) MLR coefficients with 95% confidence intervals; **b**) Bootstraps distribution of MLR R^2^; **c**) Prediction intervals for test set; **d**) Residual plot with uncertainty bands.

Bootstrap resampling confirmed that all three modeling approaches delivered extremely high predictive performance, but with different levels of robustness. The multiple linear regression (MLR) consistently outperformed the machine-learning models (Fig. 8; Table 2), yielding a mean R² of 0.9993 ± 0.0004 (95% CI: 0.9984–0.9996) and a mean absolute error (MAE) of 0.016 (95% CI: 0.010–0.031). By contrast, both Random Forest and Gradient Boosting achieved mean R² values of ~ 0.992, but with broader uncertainty ranges (95% CI: 0.984–0.998 and 0.981–0.999, respectively) and higher MAE (~ 0.043 with wider confidence intervals).

Feature importance analysis further highlighted the absolute dominance of discharge as the efficiency driver. In Random Forest, discharge explained 99.8% of the variance on average (mean importance = 0.998, 95% CI: 0.994–0.999), while all other predictors contributed marginally: gross head ~ 0.002 (CI: 0.0005–0.005), season ~ 0.0004 (CI: 0.0001–0.0011), and available capacity ~ 0.0002 (CI: <0.001–0.0005). Gradient Boosting produced an almost identical distribution of importance, fully consistent with the linear regression coefficients. Pairwise bootstrapped comparisons confirmed the statistical dominance of MLR. The average R² advantage of MLR was 0.0073 ± 0.0036 over Random Forest and 0.0071 ± 0.0045 over Gradient Boosting, with > 99.9% probability that MLR performed better in both cases. Random Forest and Gradient Boosting were statistically indistinguishable from each other (ΔR² = − 0.0001 ± 0.0035). Together, these findings show that while non-linear models reproduce efficiency variability reliably, their marginal gains are negligible relative to the parsimonious linear model, which emerges as the most robust framework for capturing the discharge–efficiency relationship.

**Table 2:** Model performance and uncertainty metrics based on bootstrap resampling.

Model	*R* ^2^_mean_	95% CI (*R*²)	MAE (mean)	95% CI (MAE)	Key Notes
Multiple Linear Regression	0.9993 ± 0.0004	0.998–0.9996	0.016	0.010–0.031	Highest accuracy and narrowest CI; stable coefficients
Random Forest	0.9921 ± 0.0039	0.984–0.998	0.044	0.030–0.058	High accuracy, but less stable; importance dominated by discharge
Gradient Boosting	0.9922 ± 0.0047	0.981–0.999	0.043	0.026–0.060	Similar to RF; slightly wider uncertainty bands

### Operational dynamics

**Fig. 9:**
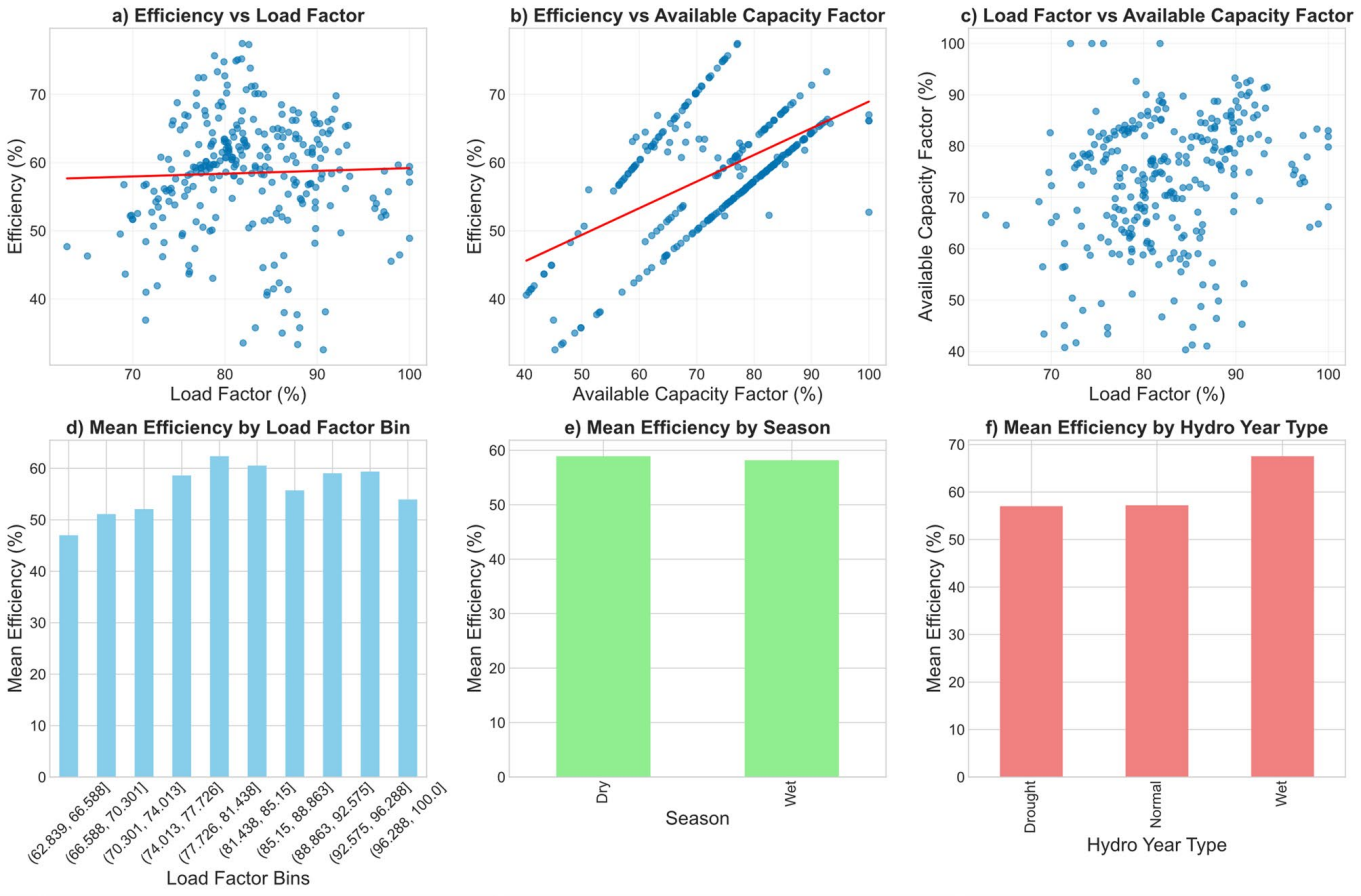
Operational performance of the Ruzizi I plant. (**a–c**) Correlation analyses between efficiency, load factor, and available capacity, (**d**) Binned analysis, (**e**) Seasonal mean efficiency, and (**f**) Efficiency by hydro-climatic year type.

The correlation analysis among operational metrics revealed distinct patterns in the internal functioning of the Ruzizi I plant. Efficiency showed a weak correlation with load factor (*r* = 0.03, *p* > 0.5), confirming that variations in plant loading do not systematically translate into proportional changes in turbine conversion performance (Fig. 9a). By contrast, efficiency correlated moderately with available capacity factor (*r* = 0.57, *p* < 0.001), suggesting that periods with higher availability of generating units tend to sustain higher efficiency levels (Fig. 9b). This relationship underscores the importance of operational readiness and turbine availability in determining energy conversion performance, beyond hydrological constraints. Moreover, the analysis revealed a very subtle correlation between the available capacity and the load factor (Fig. 9c).

When examined through binned analyses (Fig. 9d), efficiency exhibited a clear dependence on both load factor and available capacity. For load factor, efficiency increased from low values (< 65%) up to an optimal range around 78–82%, where the mean efficiency peaked at 62.4%. Beyond this zone, efficiency declined slightly, particularly under high loading (> 90%), indicating diminishing returns and possible hydraulic or mechanical constraints under near-maximum utilization. Similarly, efficiency rose steadily with increasing available capacity, reaching average values above 65% when availability exceeded 88–90%. This reflects the stabilizing effect of redundancy and operational reserves on conversion performance. Statistical testing reinforced these findings (Table 3). Linear regression between efficiency and load factor yielded a negligible fit (R² = 0.001), whereas the relationship with capacity factor was more robust (R² = 0.33). This confirms that while day-to-day loading patterns exert little influence, systemic availability plays a significant role in determining overall efficiency.

Seasonal stratification showed minimal differences (Fig. 9e), with mean efficiency of 58.9% in the dry season and 58.2% in the wet season, suggesting that seasonal inflow variability alone does not govern conversion efficiency. However, when grouped by hydro-climatic year types (Fig. 9f), clear contrasts emerged: efficiency averaged only 57% during drought and normal years, but rose markedly to 67.5% in wet years. Interestingly, this improvement occurred despite lower mean load factors in wet years (80% vs. 82–87% in other classes), suggesting that abundant inflows allow turbines to operate closer to their design head and flow conditions, yielding superior efficiency.

The optimal operating point analysis identified the load factor range of 78–82% as the most favorable, delivering mean efficiency of 62.4% (Table 3). In comparison, the long-term average operation at Ruzizi I has been characterized by a mean load factor of 82.5% and an average efficiency of 58.5%. This implies a potential efficiency gain of nearly 4% points if operations could be maintained closer to the identified optimal range. These findings highlight the value of targeted operational adjustments, particularly around load management and unit scheduling, for maximizing energy yield from available resources.

**Table 3:** Summary of operational dynamics and efficiency analysis.

Analysis Component	Metric/Group	Value(s)	Notes
Correlation Analysis	Efficiency vs. Load Factor	*r* = 0.034 (*p* = 0.57)	Weak, not significant
	Efficiency vs. Capacity Factor	*r* = 0.571 (*p* < 0.001)	Moderate, significant
Regression Fits	Efficiency vs. Load Factor	Eff. = 55.12 + 0.041×LF (R² = 0.001)	No explanatory power
	Efficiency vs. Capacity Factor	Eff. = 29.85 + 0.391×CF (R² = 0.327)	Stronger explanatory link
Seasonal Means	Dry season	Eff. = 58.9%, LF = 82.9%, CF = 73.0%	Nearly identical to wet
	Wet season	Eff. = 58.2%, LF = 82.2%, CF = 73.4%	
Hydro-Climate Class Means	Drought years	Eff. = 57.0%, LF = 86.6%, CF = 73.6%	Lower efficiency, high LF
	Normal years	Eff. = 57.2%, LF = 82.3%, CF = 73.2%	Baseline
	Wet years	Eff. = 67.5%, LF = 80.0%, CF = 72.6%	Higher efficiency despite lower LF
Optimal Operating Point	Load Factor Range	78–82%	Identified efficiency peak
	Mean Load Factor (optimal)	79.6%	
	Achieved Efficiency	62.4%	Peak performance
Comparison with Average Operation	Historical average	LF = 82.5%, Eff. = 58.5%	Slightly above optimal LF
	Potential gain	+ 3.9%-points efficiency	If operations shifted closer to optimal range

The table presents correlation and regression results linking efficiency to load and capacity factors, seasonal and hydro-climatic class means, identification of the optimal load factor range, achieved peak efficiency, and potential gains relative to historical average operation.

## Discussion

### The efficiency paradox: reconciling increased generation with competing hydraulic trends

While the Ruzizi I hydropower plant has achieved a significant increase in energy output over the past two decades, primarily driven by a substantial rise in water availability, the underlying drivers of plant efficiency reveal a more complex and critical narrative. Our analysis demonstrates that the gains in energy generation have occurred alongside a persistent decline in gross head, creating a fundamental hydraulic trade-off. This study moves beyond established climate-energy correlations^3^ to deconstruct the operational efficiency of a run-of-river system reliant on a large natural reservoir. We reveal that the plant’s efficiency dynamics are not primarily governed by short-term climatic signals but are dominated by an intricate interplay between increasing flow and decreasing head, a paradox where the beneficial force of volume has thus far masked the detrimental erosion of hydraulic driving force. This finding challenges the conventional treatment of efficiency as a static parameter in hydropower assessments and underscores the necessity of integrated, long-term performance monitoring for aging infrastructure in a changing climate.

### Dominance of flow and the masking effect on head loss

The near-perfect correlation between discharge and plant efficiency (*r* = 0.998) demonstrates that Ruzizi I operates as a profoundly flow-limited system^50^, where energy conversion is overwhelmingly sensitive to water throughput. This finding reveals a critical masking effect: the substantial and statistically significant increase in turbined volume has effectively concealed the detrimental impact of the concurrent decline in gross head on overall plant efficiency. The result is a counterintuitive efficiency paradox where significant head loss (− 0.201 m/decade) does not manifest as a decline in efficiency metrics precisely because of the compounding positive influence of flow. This phenomenon can be explained by the fundamental hydropower equation, where power output is a function of both head and flow ($\:P\:=\:\eta\:\rho\:gQH$); at Ruzizi I, the positive trend in Q has dominated over the negative trend in H. However, this precarious balance underscores a growing vulnerability.

The weak negative correlation between discharge and gross head (*r* = − 0.099, Fig. 6) further reflects operational and hydraulic decoupling at the plant. This likely results from dispatch strategies that prioritize stable output, the strong buffering from Lake Kivu, and potential mismatches between inflow seasonality and electricity demand. While such decoupling helps stabilize monthly generation, it also means that high-flow periods rarely coincide with optimal head conditions—a dynamic that can dampen the plant’s long-term global efficiency, which is sensitive to the joint distribution of *Q*(*t*) and *H*(*t*)^9^. Thus, although head contributes minimally to monthly efficiency fluctuations in our analysis, its persistent decline—especially if asynchronous with high discharge—represents a latent constraint on aggregate energy conversion.

The observed rise in tailwater level, a likely driver of head loss, is consistent with downstream geomorphic changes or sediment deposition, a well-documented challenge for aging run-of-river infrastructures that threatens long-term performance and requires active management^8^. The system’s efficiency is thus intrinsically linked to sediment dynamics, suggesting that current gains are potentially unsustainable without interventions to address the root cause of head erosion.

### Asymmetric resilience and the buffering capacity of lake Kivu

The efficiency response of Ruzizi I to hydro-climatic extremes reveals a system characterized by profound asymmetric resilience. The statistical indistinguishability of efficiency during drought and normal years, contrasted with a marked 18% boost during wet years, demonstrates that the primary impact of climate variability is not one of vulnerability to loss but of opportunity for gain. This asymmetry is a direct consequence of the immense buffering capacity of the upstream Lake Kivu reservoir, which acts as a hydrological capacitor, dampening high-frequency precipitation pulses and maintaining stable outflows during dry periods^10,11^. This finding aligns with the broader understanding that large storage bodies can significantly mitigate drought impacts on downstream water resources and energy systems^2,51^. Consequently, the plant’s efficiency is effectively decoupled from seasonal rainfall patterns and short-term meteorological noise, a phenomenon further evidenced by the absence of a significant seasonal efficiency cycle. Instead, the system integrates climate signals over longer timescales, transforming episodic wet periods into sustained multi-month efficiency gains. This result challenges simplistic narratives of climate vulnerability that often assume linear, symmetric impacts and highlights a critical nuance for energy planning: for well-buffered systems, climate adaptation strategies might focus less on mitigating drought losses and more on capitalizing on the significant generation opportunities presented by wet periods.

### Operational constraints: the decoupling of efficiency from load management

Beyond hydrological drivers, the plant’s operational strategy emerges as a significant factor influencing efficiency, yet in counterintuitive ways. The weak correlation between efficiency and load factor (*r* = 0.034, *p* = 0.57) indicates that efforts to maximize power output by operating near peak capacity do not systematically enhance, and may even slightly impair, conversion efficiency. This suggests that the turbines may frequently operate outside their best efficiency point (BEP) when pushed to maximum output, a known phenomenon in turbine performance curves where efficiency drops at very high loading^52^. This is further evidenced by our binned analysis, which identified a distinct optimal load factor range (78–82%) that delivers higher average efficiency than the plant’s historical average of 82.5%. The stronger correlation with available capacity factor (*r* = 0.571, *p* < 0.001) underscores that having generating units available is a prerequisite for high efficiency, but the declining trend in available capacity factor (− 5.5%/decade) signals a critical constraint. This decline is likely indicative of increasing mechanical downtime, aging infrastructure, or grid-related curtailments that prevent the plant from fully utilizing the available hydrological resource^53^. Consequently, the plant appears caught in a sub-optimal operational regime: sacrificing peak efficiency for higher output volume while simultaneously being hampered by non-hydrological constraints that limit its ability to capitalize on the increasing water volumes. This highlights a clear opportunity for operational optimization through revised unit commitment strategies aimed at the identified optimal load range, which could yield substantial gains in energy generation from the existing resource base.

### Model performance: the supremacy of parsimony in a physically constrained system

**Fig. 10:**
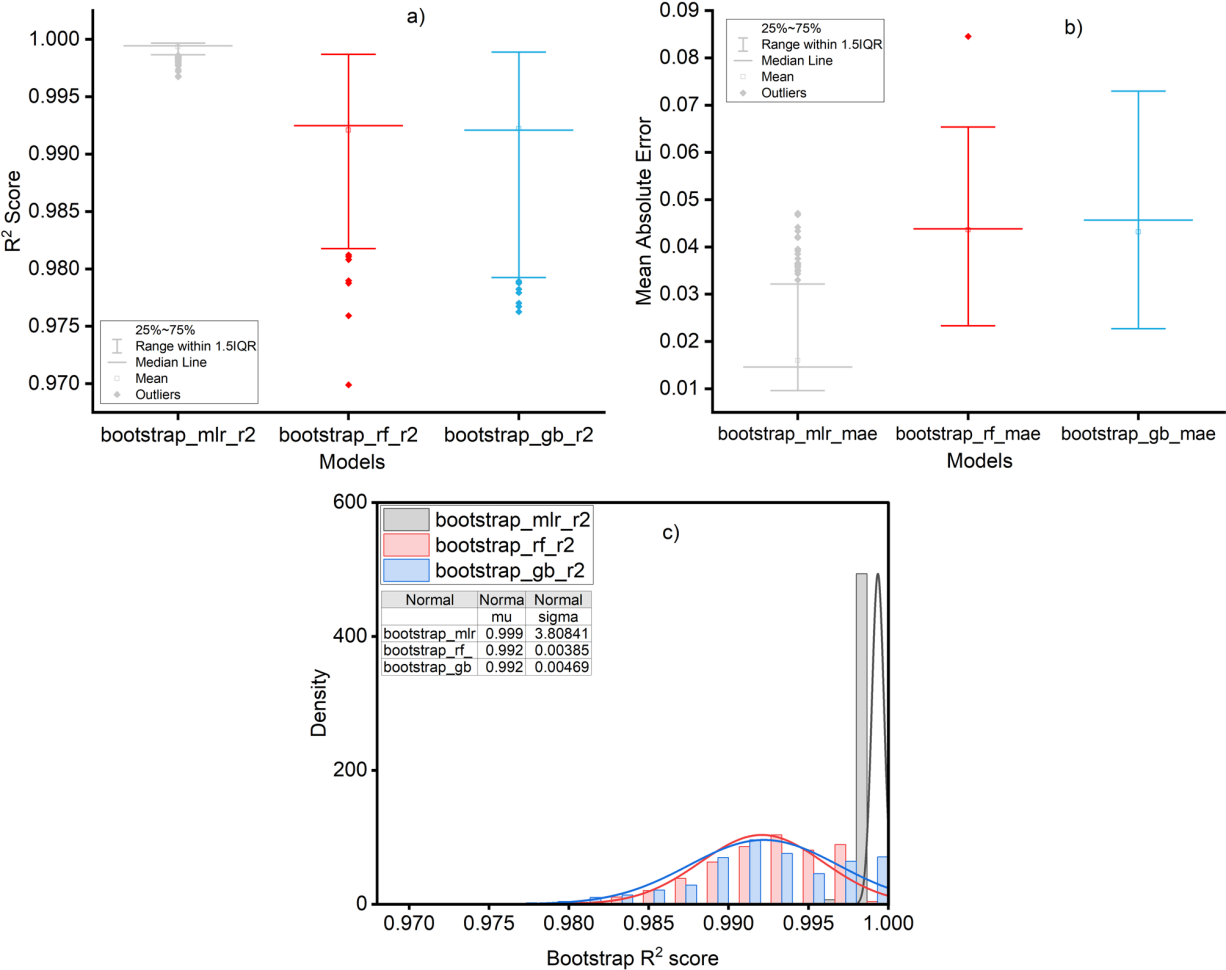
Model performance metrics: (**a**) coefficient of determination (R²), (**b**) mean absolute error (MAE), and (**c**) bootstrap distributions with 95% confidence intervals.

A particularly insightful finding of this study is the superior performance of the Multiple Linear Regression (MLR) model over the more complex Random Forest and Gradient Boosting algorithms (Fig. 10). The MLR model not only achieved a near-perfect fit (R² = 0.999) but also did so with significantly narrower uncertainty bounds and greater stability across bootstrap iterations. This result defies the common assumption that complex, non-linear machine learning models are inherently superior for predicting environmental systems (e.g^54^.,.

Instead, it strongly indicates that the relationship between the dominant predictor (discharge) and plant efficiency is inherently linear and deterministic at the monthly timescale. The physical explanation for this is rooted in the fundamental engineering of hydropower generation: for a given plant configuration and head, the conversion of hydraulic energy to electrical energy is a direct, linear function of flow rate, as reflected in the power equation. The stunning predictive power of the MLR model suggests that the efficiency calculation at Ruzizi I is essentially a mathematical function of flow, with other factors providing negligible explanatory power once discharge is accounted for. This finding underscores a critical principle in scientific modeling: parsimony. When a simple, interpretable model based on first principles captures the system’s behavior, it is preferable to a “black box” algorithm, as it enhances understanding and generalizability^55,56^. The application of ML models, while valuable for identifying this very linearity, provided no meaningful gain in predictive accuracy, demonstrating that in this specific case, the system’s physics are adequately and most robustly captured by a linear approximation.

### Synthesis, limitations, and future directions

**Fig. 11:**
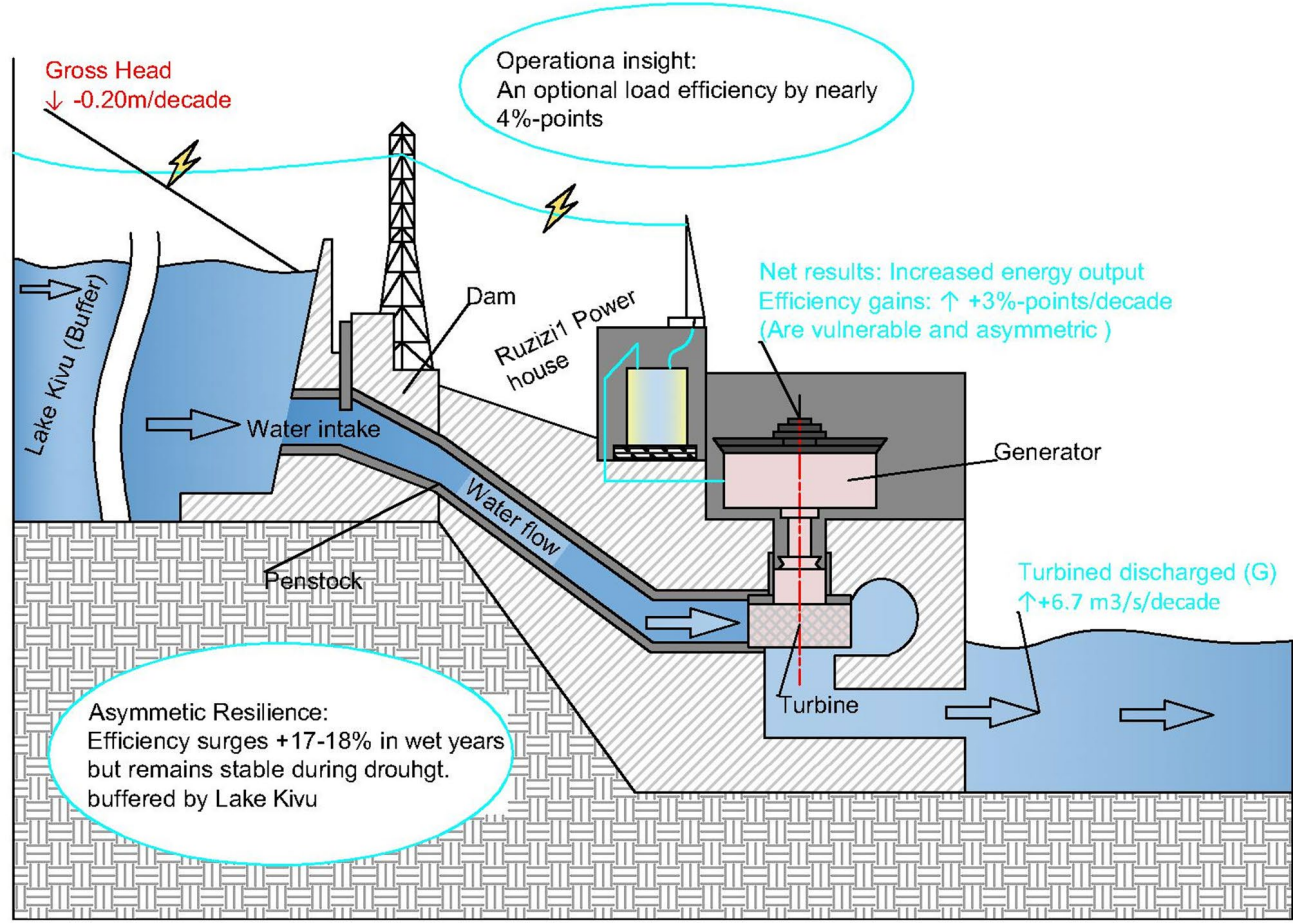
Conceptual model of the tripartite interplay governing efficiency dynamics at Ruzizi I hydropower plant (2000–2023).

Synthesizing these insights, the performance of Ruzizi I is governed by a tripartite interplay of competing forces: (1) a beneficial long-term increase in water volume, (2) a detrimental long-term decline in hydraulic head, and (3) emerging non-hydrological constraints related to aging infrastructure and grid integration. The net effect has been a rise in energy output, but this masks an evolving vulnerability where efficiency gains are increasingly reliant on high-flow conditions and may be undermined by persistent head loss and technical availability issues. This conceptual illustration is summarized in Fig. 11. A primary limitation of this study is its reliance on operational data, which, while comprehensive, does not allow for the precise attribution of the observed head loss to specific mechanisms such as climate-induced hydrological changes, sediment deposition, or anthropogenic regulation. Future research should employ a fully coupled hydrological-hydrodynamic-sediment transport model of the Lake Kivu–Ruzizi system to partition these drivers. Furthermore, the insights from the operational analysis, particularly the identified optimal load factor range, should be tested through real-time optimization simulations to quantify potential energy gains.

## Conclusion and practical recommendations

### Conclusion

This study demonstrates that the historical efficiency of the Ruzizi I hydropower plant has been overwhelmingly governed by a single variable: discharge. Our application of a multi-model machine learning framework quantitatively confirmed that month-to-month variability in efficiency is almost entirely explained by turbined volume (*r* = 0.998), while the role of gross head is statistically negligible. This finding resolves the apparent paradox of rising output amid declining head, revealing that efficiency gains are not merely compensating for but are effectively masking the erosion of the hydraulic driving force through a reliance on increased water throughput. The system’s resilience is further evidenced by its asymmetric efficiency response, remaining stable during drought but surging significantly during wet years, a buffer provided by Lake Kivu. However, this flow-dependent regime is increasingly threatened by non-hydrological constraints, as signaled by the persistent decline in available capacity factor (− 5.5%/decade). Ultimately, the long-term sustainability of this vital energy asset depends on addressing two fronts: managing the root causes of sediment-induced head loss to preserve hydraulic potential, and implementing operational strategies, such as targeting the identified optimal load factor range of 78–82%, to maximize energy conversion from the existing water endowment. This shift from maximizing output to optimizing efficiency is paramount for safeguarding the plant’s generating potential against the vulnerabilities uncovered in this diagnosis.

### Practical recommendations for stakeholders

The findings of this study translate into clear, actionable recommendations for key stakeholders responsible for the operation and planning of the Ruzizi I hydropower plant and the regional energy system.

1. For Plant Operators: Shift operational schedules towards the identified optimal load factor range of 78–82%. Maintaining operations within this range, rather than consistently aiming for maximum output, would leverage the same water volume to gain nearly 4% points in average efficiency. This operational adjustment represents a significant, no-cost opportunity to increase energy generation and revenue.
2. For Asset Managers: Prioritize a comprehensive investigation into sediment management strategies downstream of the dam. The persistent decline in gross head, driven by a rising tailwater level, is actively eroding the plant’s fundamental generating potential. Diagnosing the root cause (e.g., sediment deposition, geomorphic change) and implementing mitigation measures are critical capital investments to protect the long-term value of this asset.

For Regional Planners and Policymakers: Recognize that the strong buffering effect of Lake Kivu is a critical infrastructure asset for regional energy security. Its capacity to mitigate drought impacts and stabilize generation is a cornerstone of a resilient grid. Therefore, policies and actions aimed at protecting the hydrological health of the lake’s watershed, managing land use, pollution, and water quality, are direct investments in the reliability of the electricity supply for Burundi, the DRC, and Rwanda.

## Data Availability

The datasets generated and analyzed during the current study are available from the corresponding author upon request.
